# Synthesis, Properties, and Applications of Nanocomposite Materials Based on Bacterial Cellulose and MXene

**DOI:** 10.3390/polym15204067

**Published:** 2023-10-12

**Authors:** Aizhan B. Talipova, Volodymyr V. Buranych, Irina S. Savitskaya, Oleksandr V. Bondar, Amanzhol Turlybekuly, Alexander D. Pogrebnjak

**Affiliations:** 1 Department of Biotechnology, Al-Farabi Kazakh National University, Almaty 050040, Kazakhstan; talipova.aizhan@gmail.com (A.B.T.); irina.savickaya@kaznu.edu.kz (I.S.S.); 2 Department of Nanoelectronics and Surface Modification, Sumy State University, 40000 Sumy, Ukraine; oleksandr.v.bondar@gmail.com; 3 Faculty of Materials Science and Technology in Trnava, Slovak University of Technology in Bratislava, 917 24 Trnava, Slovakia; 4 National Laboratory Astana, Nazarbayev University, Astana 010000, Kazakhstan; aturlybekuly@gmail.com; 5 Aman Technologies, LLP, Astana 010000, Kazakhstan; 6 Faculty of Mechanical Engineering, Lublin University of Technology, 20-618 Lublin, Poland

**Keywords:** MXene, bacterial cellulose, nanocomposite, sensor, tissue engineering, MXene and biomolecules

## Abstract

MXene exhibits impressive characteristics, including flexibility, mechanical robustness, the capacity to cleanse liquids like water through MXene membranes, water-attracting nature, and effectiveness against bacteria. Additionally, bacterial cellulose (BC) exhibits remarkable qualities, including mechanical strength, water absorption, porosity, and biodegradability. The central hypothesis posits that the incorporation of both MXene and bacterial cellulose into the material will result in a remarkable synthesis of the attributes inherent to MXene and BC. In layered MXene/BC coatings, the presence of BC serves to separate the MXene layers and enhance the material’s integrity through hydrogen bond interactions. This interaction contributes to achieving a high mechanical strength of this film. Introducing cellulose into one layer of multilayer MXene can increase the interlayer space and more efficient use of MXene. Composite materials utilizing MXene and BC have gained significant traction in sensor electronics due to the heightened sensitivity exhibited by these sensors compared to usual ones. Hydrogel wound healing bandages are also fabricated using composite materials based on MXene/BC. It is worth mentioning that MXene/BC composites are used to store energy in supercapacitors. And finally, MXene/BC-based composites have demonstrated high electromagnetic interference (EMI) shielding efficiency.

## 1. Introduction

Multifunctional materials and structures find extensive applications in numerous fields of engineering and technology, including supercapacitors (SC), batteries, and catalysts. This is primarily due to their ability to perform multiple structural functions and possess unique features that make them highly versatile and valuable in these areas [1,2]. MXene refers to a group of two-dimensional (2D) carbides with the chemical formula M_n+1_X_n_T_x_ [3,4]. In this formula, M represents a transition metal, X represents carbon and/or nitrogen, and T_x_ represents various surface terminations, such as –OH, –O, or –F. MXene materials are known for their notable properties, including a large surface area and high electrical conductivity. They also possess characteristics such as nontoxicity and mechanical strength, making them safe and durable. Additionally, MXene materials are lightweight and exhibit excellent electromagnetic interference (EMI) shielding performance, making them suitable for applications in various fields. Furthermore, MXene materials possess a lamellar structure with numerous hydrophilic groups on their surface. This characteristic allows for the controllable conductivity of MXene by adjusting the interlayer spacing under specific pressures [5]. Additionally, ultrathin MXene nanolayers contain unbounded metal atoms and surface groups, which enhance their interaction with organic molecules [6]. Despite the great success in developing self-assembled compressible and elastic MXene macrostructures [7,8,9], it is still challenging because of weak interlayer interaction. It is known that MXene has been effectively integrated into numerous macromolecules, including proteins and carbohydrates, to produce composite materials with enhanced functionalities [10,11,12,13,14,15]. One-dimensional materials (bacterial cellulose stripes, aramid nanofibers, carbon nanotubes) have shown effective prevention of MXene repackaging and interlayer space expansion [16,17,18].

Bacterial cellulose (BC) is a polysaccharide produced by microorganisms. The main properties of BC are a high level of water absorption, permeability, porosity, flexibility, mechanical strength, biodegradability, and biocompatibility [19,20]. Being nonallergenic, safe, and able to be sterilized, BC has become an immensely popular biomaterial for medical applications [21]. Due to its transparency and superior wound site adherence, BC is frequently utilized for wound healing, especially as artificial skin in the treatment of extensive burns [22]. The swelling properties of BC-based hydrogels are caused by the hydrophilic groups presented in the polymer chains that open BC for use as bandages for wound treatment. The wound healing process accelerates by maintaining high oxygen content and a moist environment in the wound and absorbing excess tissue exudate [22]. However, the majority of BC-based bandages for wound healing simply work to rehydrate tissues better and reduce infections near the area. They seldom engage in control of the behavior of endogenous wound healing cells. Implementation of BC as a passive treatment tool results in a slow healing process of wounds. Nuccitelli et al. showed occurring differences in transepithelial potential (TEP). When the epithelium is damaged, an endogenous direct current electric field (DCEF) develops at the wound sites and lasts until the wound healing process is finished [23,24]. This physiological EF has the potential to act as the primary signal for controlling cell behaviors like adhesion, proliferation, migration, and differentiation. As a result, it is crucial in boosting regenerative activity for wound healing and the repair of damaged tissues. Exogenous electrical stimulation (ES), which mimics endogenous EF’s natural wound healing mechanism, accelerates skin regeneration. BC does not conduct electricity [25]. Therefore, a BC-based wound dressing cannot affect the behavior of electrically sensitive cells. One promising way to improve hydrogels’ electrical conductivity and mechanical characteristics is by applying functional inorganic nanoparticles to create composites. The development of a composite based on BC and MXene would solve the problems described above. Composites based on BC and MXene are excellent candidates for the treatment of wounds.

BC is super elastic and perfectly adheres to the surface of the skin. MXene is included in many composites for wearable electronics. It should be assumed that composites based on BC and MXene will meet all the requirements for wearable electronics. The use of BC/MXene-based films in wearable electronics was demonstrated by Yang et al. [16]. The developed sensor device can track a variety of human biological activities (swallowing, heartbeat and pulse pulsation), acoustic vibrations, and gestural movements [16]. According to the results of the study, the composite based on BC and M showed high mechanical accuracy (225 MPa), high sensitivity (up to 95.2 kPa^−1^, in <50 Pa region), fast response (95 ms), and outperformed repeatability (25,000 cycles) [16]. Ma et al. [26] demonstrated the existence of strong chemical adsorption and hydrogen bond interaction between BC and Ti_3_C_2_T_x_ MXene, which enables good film stabilization. The authors developed a wearable and ultrathin bacterial celluloses/MXene film with Janus structure [26]. High tensile strength (up to ∼532.87 MPa) and wrinkling (up to ∼6152 cycles) were displayed by the manufactured ultrathin BCs/MXene sheet, which had a thickness of 1.732 m. In particular, the BC/MXene film was ultralight and breathable. The bottom layer was made of BC, which made the composite skin-friendly. The fight against radiation pollution is one of the main tasks of our time. Composites based on BC and MXene are successfully used in materials for electromagnetic interference shielding [26,27,28]. Developed BC/MXene ultrathin film showed 99% electromagnetic interference shielding and achieved outstanding *SSE/t* ∼69,455.2 dB cm^2^ g^−1^ [26].

In this review, we revisited the latest advances in the fabrication of BC/MXene composites and their multifunctional applications in human activities.

## 2. Synthesis of MXene/BC-Based Composites

### 2.1. Fabrication of Multilayer Ti_3_C_2_T_x_-MXene

Numerous methods are used for MXene fabrication. The synthesis of MXene involves selectively etching several atomic layers from the layered MAX phase precursor. The MAX phase refers to ternary carbides or nitrides, with M representing transition metals, A representing interleaved atoms of elements from groups 13 and 14, and X representing carbon or nitrogen [2].

Synthesis methods can be categorized based on the type of etching agent: those containing fluorine (HF or LiF) [26] and those that are fluorine-free (employing Lewis acids or alkalis) [29,30]. Aqueous fluoride-containing acids have found widespread use as etchants for this specific purpose [31]. After the selective etching step, the resulting accordion-like multilayered structure undergoes delamination through intercalator agents and a sonication process [29,30,31]. In order to achieve intercalation and subsequent exfoliation, a range of organic compounds (such as dimethyl sulfoxide, tetrabutylammonium hydroxide, urea, hydrazine, and isopropanol), as well as inorganic ions (like Li^+^ or Na^+^), are employed. These agents enable the insertion of ions or molecules between the layers of materials characterized by weak bonds [31].

More comprehensive protocols for the synthesis of MXene were detailed by Yu. Gogotsi et al. in their publication [32], B. Anasori et al. in [33], and Zh. She et al. in [34]. In our review, our focus is on elucidating the methods of MXene synthesis that have been applied to create MXene/BC-based composites.

**Figure 1:**
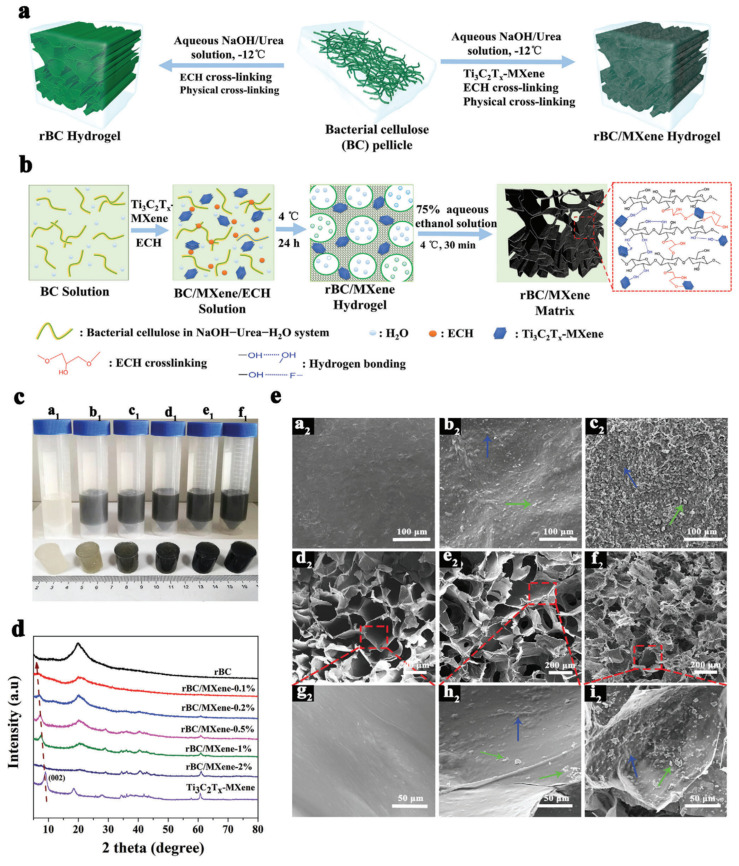
Synthesis of BC/MXene: (**a**) representation of erythrocyte-based hydrogel fabrication; (**b**) diagram of the development of BC/MXene composite hydrogels; (**c**) optical images of erythrocyte-based hydrogels (bottom) and erythrocyte-based solutions (top); (**a1**–**f1**) optical images depict the comparison between pure bacterial cellulose (BC) and BC/MXene composite samples with varying concentration of MXene. The wt.% of MXene in the composite samples are 0.1, 0.2, 0.5, 1, and 2 wt.% MXene, respectively; (**d**) X-ray patterns of erythrocyte−based hydrogels with varying MXene content; (**e**) SEM micrographs from the surface (**a2**,**b2**,**c2**), cross-section (**d2**,**e2**,**f2**) and larger magnification of SEM images showing pore walls (**g2**–**i2**) of clean BC-hydrogel, 0.1% hydrogel and 2% hydrogel, respectively. Blue arrows indicate the insertion of MXene into the bacterial cellulose (BC) matrix, while green arrows indicate the incorporation of MXene onto the surface or pore wall of BC/MXene hydrogels. Reprinted with permission from [35] 2020 John Wiley and Sons.

**Figure 2:**
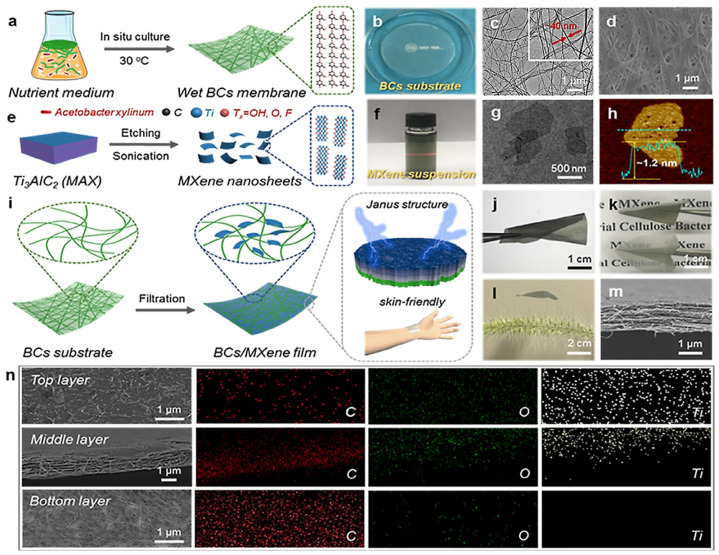
Synthesis of the BC/MXene composite material [37,38,39,54]: (**a**) wet BC membrane synthesis through in situ cultivation of *Acetobacter xylinum*; (**b**) optical image of membrane; (**c**) TEM image of BC membrane; (**d**) SEM image of BC; (**e**) d-Ti_3_C_2_T_x_ MXene nanosheets are made from MAX (Ti_3_AlC_2_) phases using HCl acid and LiF; (**f**) MXene slurry captured through optical imaging; (**g**) TEM image of d-Ti_3_C_2_T_x_ MXene nanolayers; (**h**) AFM image showcasing the morphology and topography of d-Ti_3_C_2_T_x_ MXene; (**i**) scheme of BC/MXene composite fabrication process; (**j**–**l**) optical images depicting the appearance and structure of flexible, ultrathin BC/MXene ultralight composites; (**m**) SEM cross-sectional image revealing the layered or lamellar composite film structure; (**n**) SEM image followed by EDS elemental mapping displaying BC/MXene composite with Janus structure. Adapted from [26] 2021 Elsevier.

The researchers [35] utilized a specific method to produce the multilayer Ti_3_C_2_T_x_-MXene for the BC/MXene composite. They achieved this by selectively etching the MAX phase (Ti_3_AlC_2_) using a 49% aqueous solution of hydrofluoric acid (HF). The etching process effectively removed the aluminum (Al) layers, as shown in Figure 1a. Following the fabrication process, the resulting suspension was subjected to multiple washes using deionized water. Additionally, the suspension was centrifuged until it reached a pH level 6. The resulting residue was a multilayered Ti, which was further sonicated and centrifuged to receive a homogeneous dark green suspension [35]. In the work of Ma et al. [26], the delamination of Ti_3_C_2_T_x_ (d-Ti_3_C_2_T_x_) MXene was performed by selective etching of the Al from Ti_3_AlC_2_ (Figure 2a,c). This process involved the use of hydrochloric acid (HCl) and lithium fluoride (LiF). Figure 2e illustrates the manufacturing process scheme, which outlines the various steps involved in the production of the material. To etch the aluminum layer, 1 g of Ti_3_AlC_2_, 1 g of LiF, and 20 mL of 9 M HCl were added to the mixture, and the resulting suspension was stirred for 48 h at 35° [26].

In their study, Yang et al. employed bacterial cellulose nanofibers to facilitate the intercalation of Ti_3_C_2_T_x_ MXene, thereby enabling control over the interlayer distance. The Ti_3_C_2_T_x_ multilayer nanolayers were prepared using a combination of traditional selective etching and ultrasonic treatment, as described in [16]. In this process, 1.0 g of LiF powder and 20 mL of HCl were mixed and stirred for 10 min. Subsequently, 1.0 g of Ti_3_AlC_2_ was added to the mixture. After allowing the interaction to occur at 35 °C for 24 h, the resulting material was washed with deionized water and centrifuged at 8000 rpm until the pH of the supernatant reached 6. Next, the Ti_3_C_2_T_x_ was dispersed in ethanol and subjected to sonication for two hours to obtain layered Ti_3_C_2_T_x_ nanolayers. Afterwards, the exfoliated nanolayers were centrifuged at 10,000 rpm to remove ethanol and redispersed in 60 mL of deionized water. Following sonication and centrifugation at 3500 rpm for 10 min, a stable colloidal suspension of Ti_3_C_2_T_x_ multilayer was successfully fabricated.

### 2.2. Fabrication of Bacterial Cellulose (BC)

Bacterial cellulose (BC) is a biomaterial synthesized by bacteria from the genera *Gluconacetobacter*, *Sarcina*, and *Agrobacterium*. Although many microorganisms synthesize BC, the most efficient BC producers are *Komageteibacter xylinus* (*Acetobacter*). BC synthesis begins with culturing *Komageteibacter xylinus* (*Acetobacter*) in Hestrin–Schramm (HS) medium (2% glucose, 0.5% yeast extract, 0.5% peptone, 0.27% Na_2_HPO_4_, and 1.5% citric acid) [36,37,38,39]. The cellulose producer is prepared on a modified Hestrin–Schramm medium with 0.1% beer wort and 0.5% ethanol to increase the cellulose synthesis [37,38,39]. The bacterial cellulose producer is cultivated for 6–7 days at 29–30 °C. There are two methods of cultivation—static and agitated. Bacterial cellulose films are produced in static conditions, while sphere-like cellulose particles are produced in agitated conditions [40]. The grown film was washed with deionized water for 5–7 min. After washing the BC films with deionized water to pH 6.8–7.2, they were further treated with a 1% (*w*/*v*) NaOH solution at 35 °C for 24 h. This process helped to remove impurities such as endotoxins of the producer. Some authors suggest an additional procedure for the purification of BC film from endotoxin by treatment with sodium dodecyl sulphate [41] and complex of enzyme [42]. Following NaOH treatment, the films were washed again with deionized water to adjust the pH. Finally, the BC films were sterilized by autoclaving, ensuring that any remaining microorganisms were eliminated and the films were made suitable for further use [43,44].

### 2.3. Fabrication of the BC/MXene Based Composite Films

The slow diffusion of multivalent charge carriers between MXene layers has been a long-standing issue, primarily attributed to the narrow physical space and strong electrostatic attraction [45]. The addition of a one-dimensional BC layer between MXene flakes resolves the abovementioned issue, namely, that due to hydrogen bonds and electrostatic forces, the interlayer space expands, increases dispersibility and film-forming capability, and enhances the composite’s mechanical properties, including strength, rigidity, and toughness [46].

Fabricating the BC/MXene composite presents challenges because BC is insoluble in water. This insolubility arises from intramolecular hydrogen bonding between hydroxyl groups in the BC structure. As a result, it is difficult to disperse BC evenly in water-based solutions and achieve a homogeneous mixture with MXene nanosheets. It is difficult to dissolve BC in typical organic solvents (n-hexane, tetrachloroethylene, and toluene) [47]. However, BC’s are quite beneficial in tissue engineering because of their affinity to water and limited solubility [48].

To prepare the BC/MXene composite, Mao et al. [35] took a dried BC suspension and dissolved it in the NaOH-Urea-H_2_O solution (7:12:81) under low temperature according to Jiang et al.’s protocol [49]. To prepare the BC/MXene composite, freshly prepared Ti_3_C_2_T_x_-MXene was added to the BC solution. Different concentrations of MXene were incorporated into the BC solution, resulting in BC/MXene mixtures with 0.1%, 0.2%, 0.5%, 1%, and 2% MXene content. Introducing Ti_3_C_2_T_x_-MXene into the BC solution makes it possible to create BC/MXene composite hydrogels with varying MXene content. This is achieved through a combination of chemical covalent and physical crosslinking mechanisms. Chemical covalent crosslinking occurs between a significant number of hydroxyl (–OH) groups present in the cellulose chains. This crosslinking is facilitated by using epichlorohydrin (ECH) as a crosslinking agent. On the other hand, physical crosslinking results from hydrogen bonding and self-entanglement of chains between cellulose chains, as well as the hydration of cellulose II crystallites [50]. A 75% aqueous ethanol solution is added to the mixture to initiate the crosslinking process and form the hydrogels. This combination of solvents aids in the formation of BC/MXene composite hydrogels with the desired concentration of MXene (Figure 1a,b) [35].

Optical images presented in Figure 1c depict BC-based hydrogels with varying weight percentages of MXene. The MXene concentrations were 0, 0.1, 0.2, 0.5, 1, and 2 wt.%. The color of the hydrogels gradually darkens with increasing MXene content. The XRD patterns in Figure 1d demonstrate that the pure BC hydrogel exhibits a faint peak at 12.1° and distinct diffraction at 20.0°. These peaks correspond to the (110) and (200) reflections of the cellulose II crystal planes, respectively [51,52]. Contrarily, the X-ray diffraction patterns of BC/MXene composite hydrogels demonstrate a significant decrease in the intensity of the (110) and (200) BC peaks. Simultaneously, there is a gradual increase in the characteristic peaks (001) of MXene as the MXene content in the hydrogels increases. Furthermore, the (002) peak of Ti_3_C_2_T_x_-MXene in BC/MXene hydrogels exhibited a shift towards a lower two-angle level. This shift suggests a slight increase in the d-spacing of MXene within the BC/MXene hydrogels, providing strong evidence of the successful integration of MXene into the hydrogel structure [53]. In Figure 1e, one can observe freeze-dried SEM images depicting the BC/MXene composite hydrogels. These images reveal that the content of MXene has a discernible impact on the microstructure of BC, resulting in the formation of an asymmetric structure. This structure consists of an upper surface layer and a porous, spongy layer, both exhibiting a three-dimensional interconnected arrangement. Furthermore, the authors have detected the presence of elements including C, Ti, O, and F (as depicted in Figure S1), which are likely attributed to the presence of MXene and still retain fluorine terminals.

In the study conducted by Yang et al. [16] and Ma et al. [26], composite films were fabricated through a vacuum filtration method. To create a suspension of MXene d-Ti_3_C_2_T_x_ nanolayers, a low concentration of 0.005 mg∙mL^−1^ was achieved by diluting with deionized water. To prepare the BC/MXene films, the MXene suspension was subjected to multiple vacuum filtration cycles on a BC substrate. During each filtration cycle, 10 mL of the MXene dispersion was used. After filtration, the BC/MXene films were left to dry at room temperature for an hour [26].

Ma et al. proposed obtaining BC/Mxene films with a Janus structure using vacuum filtration, and not by simple mixing [26].

Due to its porous structure (Figure 2d), BC can capture MXene nanosheets.

The BC film membranes were produced by *Acetobacter xylinum* (Figure 2a). Next, the BC films are washed and purified. Figure 1b shows a transparent BC film.

The synthesis and key features of resulting material could be generalized as follows: The incorporation of MXene nanosheets was accomplished by exploiting the porous structure of the BC nanofibers, as illustrated in Figure 2c,d. Through the etching of MXene nanosheets (d-Ti_3_C_2_T_x_), a uniform colloidal dispersion was successfully achieved, which exhibited a typical Tyndall scattering effect, as depicted in Figure 2e,f. Synthesis of 2D d-Ti_3_C_2_T_x_ nanosheets, with an average thickness of approximately 1.2 nm, was confirmed via Figure 2g,h, substantiated using X-ray diffraction analysis. The vacuum filtration process facilitated the combination of BC and d-Ti_3_C_2_T_x_ compounds, as seen in Figure 2i–l, ultimately resulting in the formation of the characteristic lamellar structure observed in the BC/MXene hybrid film (Figure 2m), characterized by its Janus-type configuration (Figure 2n). Consequently, the thickness of the BC membrane experienced a significant reduction, while the integration of MXene nanosheets into BC films led to the creation of a dried ultrathin BC/MXene film.

In summary, it can be concluded that the BC is a substrate for supporting MXene nanosheets and serving as a scaffold for immobilizing permeable MXene nanosheets. A notable observation was that the pores within the BC membrane were distributed in three dimensions. Additionally, the MXene nanosheet was unable to completely penetrate the BC membrane, resulting in the Janus structure formation. The water gradually left the membrane in continuous filtration and drying of the BC membrane.

## 3. Physical–Chemical Properties of Composite BC/MXene Films

The structure of titanium exposed to high-frequency etching Ti_3_C_2_T_x_-MXene powder exhibits a multilayered, stacked arrangement and easily conforms to uniform layers. The XRD diagram reveals that the most intense peak at 39°, corresponding to the MAX phase (Ti_3_AlC_2_), disappears after HF etching. Additionally, the (002) peak at 9.5° and the (004) peak at 19.4° in the MAX phase exhibit broadening and shifting to 8.8° and 18.5°, respectively. Therefore, based on these observations, the authors concluded that Ti_3_AlC_2_ was effectively converted to Ti_3_C_2_T_x_-MXene and the aluminum (Al) element was replaced by hydroxyl (–OH) or fluorine (–F) fragments [55]. The XPS and high-resolution XPS analyses further supported the successful synthesis of multilayer Ti_3_C_2_T_x_-MXene. These analyses revealed that the synthesized MXene terminated with functional groups such as oxygen (–O), hydroxyl (–OH), and fluorine (–F).

**Figure 3:**
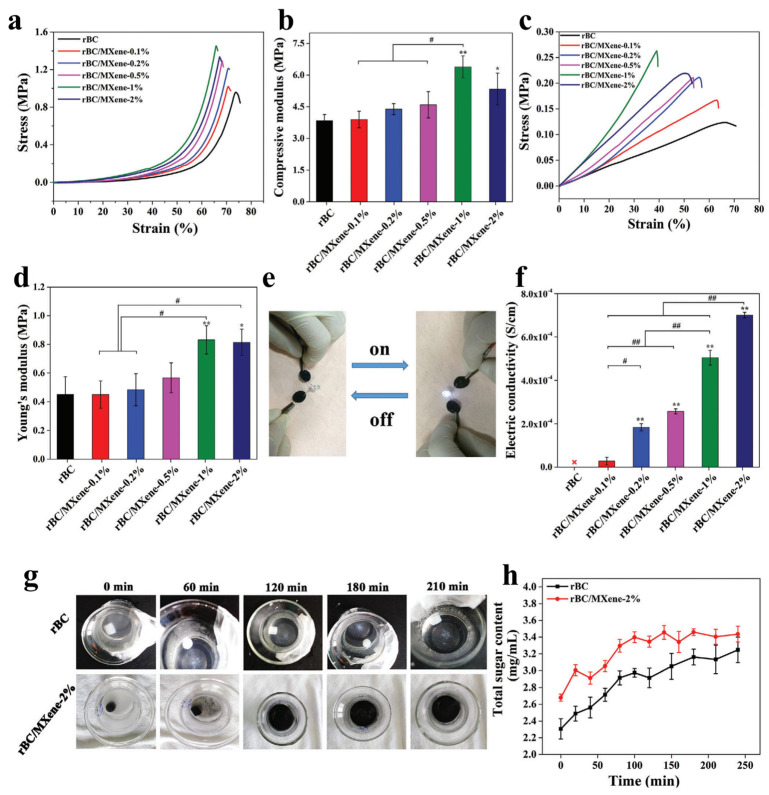
Chemical and physical characteristics of BC and BC/MXene: (**a**) compressive stress–strain curves; (**b**) compressive modulus; (**c**) curves of tensile stress against strain; (**d**) elastic modulus of BC/MXene hydrogels; (**e**) status of the LED attached to the BC/MXene 2%; (**f**) measured conductivity of hydrogels based on erythrocyte; (**g**) optical images; (**h**) the total sugar content of the cellulase solution that was measured after degrading BC and BC/MXene 2% hydrogels with cellulase at different time intervals. Data are depicted as mean ± SD (*n* = 5), and one-way ANOVA with Tukey’s multiple comparison test was applied for determination of significant difference. * *p* < 0.05; ** *p* < 0.01, as compared to the pure rBC hydrogel group; # *p* < 0.05; ## *p* < 0.01, as compared to the rBC/MXene hydrogels with different content of MXene. Reprinted with permission from [35] 2020 John Wiley and Sons.

Figure 3 displays the mechanical properties of BC-based hydrogels. It is noteworthy that the compressive stress and tensile stress at break (Figure 3a,c), as well as the compressive modulus and Young’s modulus (Figure 3b,d), exhibited a gradual increase when the MXene content was 1% or lower. The decrease in compressive and tensile strength, as well as the compressive modulus, of the hydrogels with a higher MXene content (2% and above) can be attributed to two factors. Firstly, in BC/MXene low MXene hydrogels, the mechanical strength increases due to the reinforcing effect of Ti_3_C_2_T_x_-MXene and the crosslinking reaction between the –OH groups on the MXene and BC chains. Additionally, van der Waals physical forces and intermolecular hydrogen bonds are formed between the negatively charged end groups (i.e., –F, –O, and –OH) on the surface of MXene and the numerous –OH groups on BC cellulose chains. However, in BC/MXene hydrogels with higher MXene content, the large concentrations of Ti_3_C_2_T_x_-MXene particles within a highly viscous solution can disrupt the original bonds between BC chains or between BC and MXene chains. This disruption can lead to disruption of chemical and physical crosslinking, including hydrogen bonds. Additionally, the high MXene concentration can cause some rearrangement of the chains, ultimately resulting in a decrease in mechanical strength [35].

The fabricated materials exhibit significantly improved characteristics compared to pure BC hydrogels. Moreover, the introduction of MXene has been found to enhance the mechanical strength and antifriction performance of BC/MXene composite hydrogels [56]. Furthermore, BC/MXene hydrogels possess the ability to revert back to their original state after being compressed. In the strain–stress plot of BC/MXene 2% hydrogel (Figure 3a), it can be observed that when different maximum strains are applied, each unloading path from 10% to 50% strain concludes at nearly the same point where reloading takes place. This indicates the excellent recoverability and flexibility of BC/MXene hydrogels.

**Figure 4:**
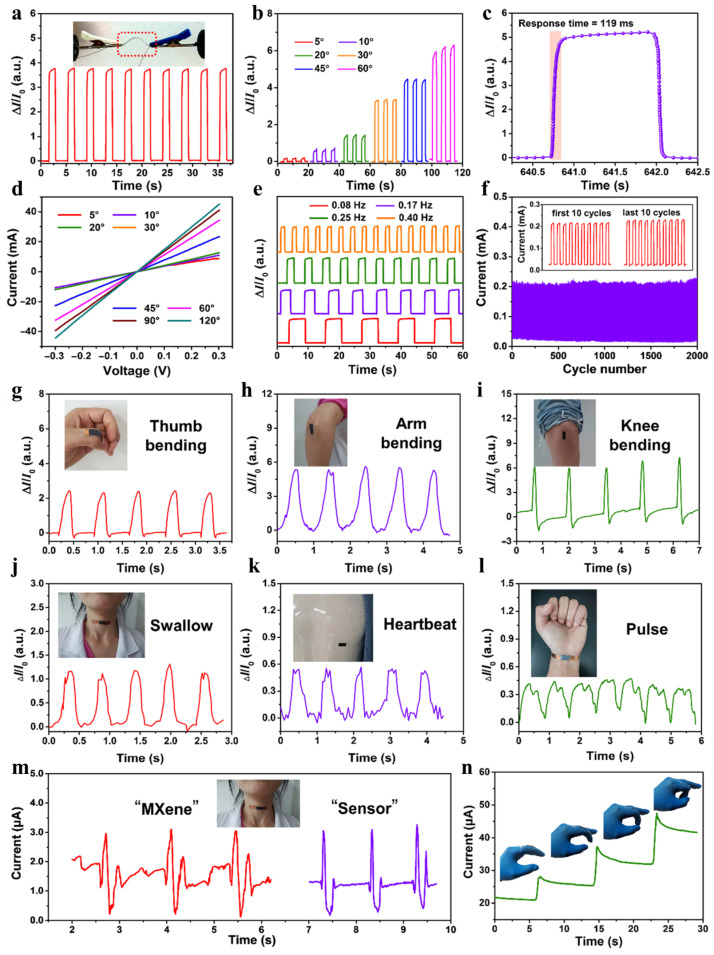
TB pressure sensor features under various bending conditions and human movements, including subtle pressures such as swallowing, heartbeat, pulse, bending deformations, and acoustic vibrations. (**a**) I-t curves in real time in a repeated bending–extension process, in the insert is an image of the sensor at bending angle 45°; (**b**) current–time dependence on the bending angle, ranging from 5° to 60°; (**c**) bent state sensor response time; (**d**) current–voltage curves at different bending angles 5–120° (for angle of 30° it was overlapped by 20°); (**e**) I-t graphs of the pressure sensor in real time at various frequencies at which bending and extension occur for a bending angle of 45°; (**f**) TB pressure sensor’s repeatability for 2000 flexion–extension cycles at 0.17 Hz with a 45° bending angle is demonstrated by the I-t curves of the first and last ten cycles on the inserts; (**g**) curves of the finger movement in real time; (**h**) curves of the hand movement in real time; (**i**) curves of the knee movement in real time; (**j**) curves of the swallowing in real time; (**k**) curves of the heartbeat in real time; (**l**) I-t graphs for pulse; (**m**) acoustic vibrations from pronouncing “MXene” and “sensor”, respectively; (**n**) real-time curves of the current time as fingers are bent in a continuous way. Adapted from [16] 2021 Springer.

The TB pressure transducer was assessed in a flexion–extension process, demonstrating rapid current response and recovery at a 45° bend angle (Figure 4a). Current characteristics improved with increasing bend angles (Figure 4b). The sensor exhibited a fast 119 ms response to mechanical load (Figure 4c) and stable sensitivity under static bending stress (Figure 4d). At a 45° bend angle, it maintained stable current response within a frequency range from 0.08 to 0.4 Hz (Figure 4e). In a cyclic test, the sensor displayed excellent reproducibility and minimal current fluctuations during 2000 cycles (Figure 4f) [16].

The TB pressure sensor’s low-pressure sensing capabilities make it ideal for detecting human movements. It was placed on various body parts (Figure 4g–n) with polyimide tape for insulation. The sensor showed fast and stable current responses during finger, arm, and knee movements (Figure 4g,i). It can capture delicate biomedical signals like heartbeats and pulses (Figure 4g,k,l) and is sensitive to acoustic vibrations (Figure 4m). Furthermore, it can monitor pressure, bending, and acoustic forces with exceptional sensitivity, stability, and versatility (Figure 4n) [16].

**Figure 5:**
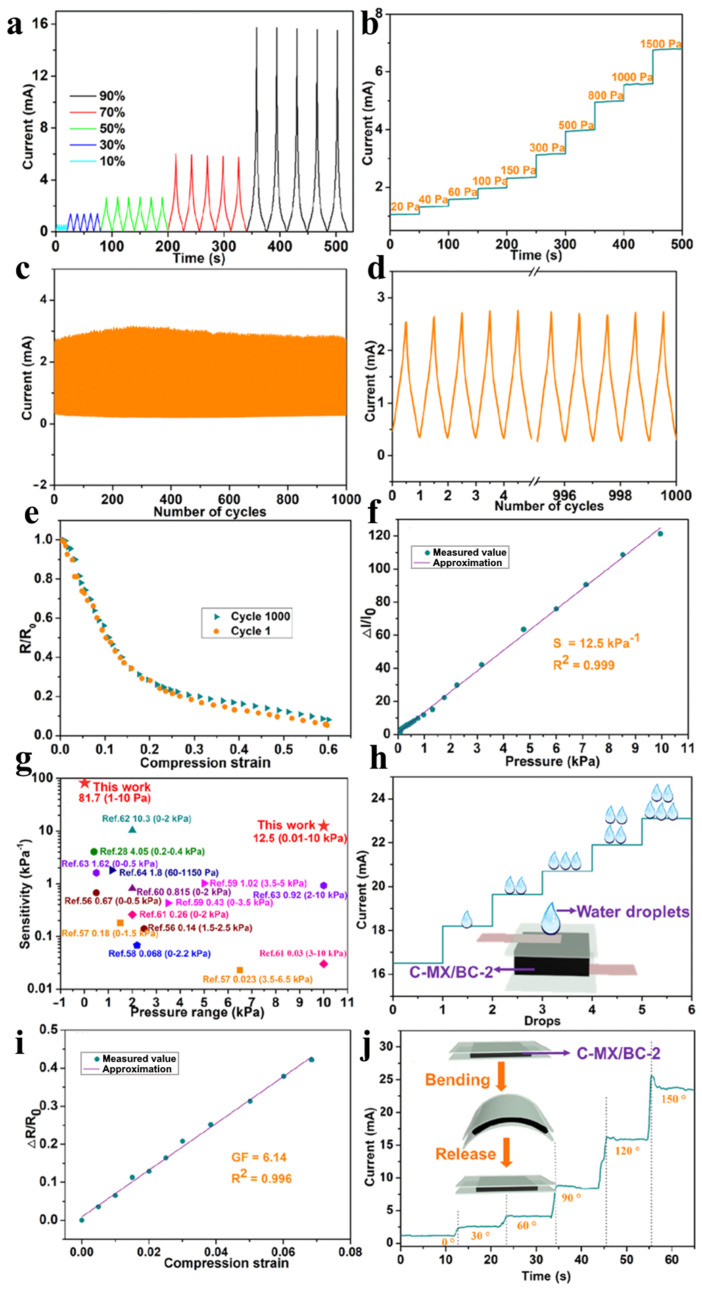
The responses to strain–current, pressure–current, and sensitivity of TB pressure sensor: (**a**) response to different compression strains; (**b**) pressure response of the current in the range of 20 to 1500 Pa; (**c**) the stability of current for 1000 cycles at 50% strain; (**d**) current at 50% strain recorded for the first and last five cycles; (**e**) *R*/*R*_0_ at 50% strain per 1000 cycles; (**f**) linear sensitivity range spanning from 0 to 10 kPa; (**g**) C-MX/BC-2 sensitivity in comparison to other sensitive materials; (**h**) current response to water drops; (**i**) the calibration factor when strain is less than 6.8%; (**j**) responses of the current to different bending angles. Reprinted with permission from [57] 2019 American Chemical Society.

MXene nanosheets and BC each have significant roles in creating a structured microarchitecture and uninterrupted texture, facilitating the achievement of structural engineering. Figure 5 presents the electrical characteristics of carbon aerogels, focusing on the current response of C-MX/BC-2 under varying compressive strains. The current rapidly increased during compression and decreased upon release, indicating high sensitivity to deformation. C-MX/BC-2 effectively detected different levels of compressive strain from 10% to 90%. The current also correlated with applied pressure, showcasing strong pressure–current responsiveness (Figure 5b). After one thousand cycles of 50% deformation, the current remained stable (Figure 5c,d). Normalized electrical resistance (*R*/*R*_0_) decreased linearly with strains below 15%, maintaining stability even after one thousand cycles (Figure 5e). This stability can be attributed to the hydrogel’s elastic and flexible carbon layers [16].

The C-MX/BC-2 sensor, however, stands out with its impressive linear sensitivity of 12.5 kPa^−1^ across 0 to 10 kPa (Figure 5f), backed by a remarkable coefficient of determination (*R*^2^ = 0.999). It excels in providing precise output over a broad range of compression strains (0–95%). This exceptional sensitivity and linearity are attributed to two key factors: its high-compression multilayer structure, accommodating various pressures and strains, and the utilization of flexible wave-shaped carbon plates that increase contact area proportionately to applied pressure or deformation.

Compared to existing carbon aerogels and flexible carbon films, C-MX/BC-2 surpasses in terms of high and broadband linear sensitivity (Figure 5g). Additionally, it exhibits a high gauge factor (GF) and responsiveness to even minute pressures and strains, making it exceptionally versatile for various sensor applications (Figure 5h). Moreover, the sensor’s ability to bend and generate real-time current proportional to the degree of bending underscores its multifunctional utility in strain detection. These qualities position C-MX/BC-2 as an ideal choice for diverse sensor applications (Figure 5i,j) [57].

Despite technological advancements, carbon-based pressure sensors still face a significant challenge in maintaining a high level of linearity across a wide range of pressures [58,59,60,61,62].

**Figure 6:**
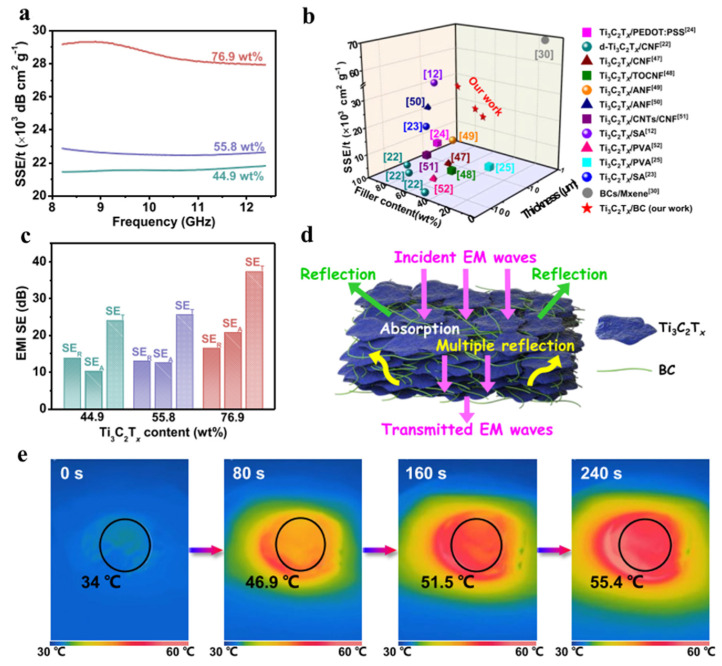
(**a**) EMI *SSE/t* Ti_3_C_2_T_x_/BC depending on the frequency; (**b**) EMI *SSE/t* for Ti_3_C_2_T_x_/BC (red stars) and different Ti_3_C_2_T_x_/polymers; (**c**) *SE_T_*, *SE_A_*, and *SE_R_* for Ti_3_C_2_T_x_/BC composites; (**d**) schematic diagram illustrating the mechanism of electromagnetic interference (EMI) protection in Ti_3_C_2_T_x_/BC system; (**e**) IR images displaying the change in temperature of Ti_3_C_2_T_x_/BC-5 composite after sunlight exposure. Adapted from [27] 2021 American Chemical Society.

The Ti_3_C_2_T_x_/BC hydrogels’ electromagnetic interference (EMI) shielding efficiency (SE) was evaluated in the 8.2–12.4 GHz range (Figure 6a), with varying Ti_3_C_2_T_x_ content. Higher Ti_3_C_2_T_x_ content led to improved EMI shielding performance due to increased electrical conductivity. At 44.9 wt.% Ti_3_C_2_T_x_, the average EMI SE exceeded 20 dB, suitable for industrial use [27]. A higher content of 76.9 wt.% achieved an EMI SE of 37.3 dB. Ultra-thin Ti_3_C_2_T_x_/BC films (4.0 to 6.7 µm thick) exhibited outstanding EMI SE, crucial in applications like telecom, military, and aerospace [53,63,64]. Although increasing thickness is a viable method for enhancing EMI performance, the associated drawbacks, such as increased weight and cost, may hinder the widespread utilization of these materials.

EMI shielding depends on factors like material conductivity, thickness, and density. Ti_3_C_2_T_x_/BC-5 had an impressive SSE/t value of 29,141 dB cm^2^∙g^−1^ at just 4 µm thickness, surpassing other Ti_3_C_2_T_x_/polymer composites (Figure 6b). These films owe their excellent shielding properties to high conductivity and network structure [53,65,66,67,68,69,70,71,72,73].

Parameters such as SET, SEA, SER, and multiple reflections were assessed. Composite films showed absorption and reflection mechanisms contributing to EMI shielding, with absorption being prominent at high Ti_3_C_2_T_x_ concentrations (Figure 6c). If the *SE_T_* value is greater than 15 dB, the *SE_M_* can be disregarded and is not represented in Figure 6c [27]. Ti_3_C_2_T_x_/BC films efficiently absorbed electromagnetic waves, making them promising for light-to-heat conversion [27].

Under simulated sunlight, Ti_3_C_2_T_x_/BC-5 rapidly reached a stable temperature of 55.4 °C, confirming its exceptional electromagnetic wave absorption and EMI SE (Figure 6e). These findings underscore Ti_3_C_2_T_x_/BC’s potential for high-performance EMI shielding applications [27].

Wearable nanofilm-based materials require robust mechanical properties, and these properties tend to improve with increased filtration cycles. The BX-5 composite stands out with impressive strength (532.87 MPa) and toughness (31.14 MJ∙m^−3^), making it one of the most durable flexible MXene-based films available (Table 1) [26]. Additionally, it boasts a high elastic modulus of approximately 8.26 GPa, attributed to the three-dimensional network structure of the BC membrane, forming strong connections with the 2D MXene nanolayers [26].

Wearable functional materials based on MXene often exhibit a limiting black and opaque appearance, restricting their practical utility [26]. However, researchers have developed a method to create transparent composite films from MXene-carbon nanotubes through a complex layer-by-layer centrifugal sputtering process, albeit suitable primarily for specific cases of large-scale production and industrial protective film applications.

**Figure 7:**
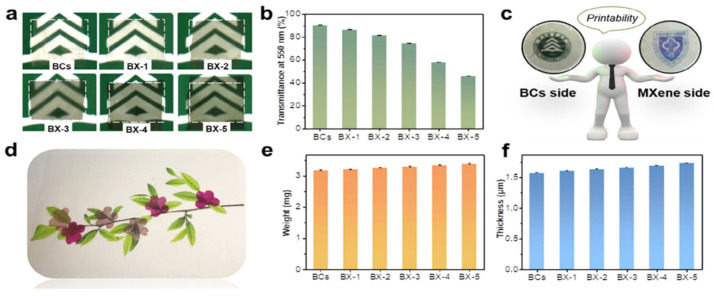
Printability and optical transparency of BC/MXene composites: (**a**) images of BC/MXene; (**b**) BC/MXene transmittance at 550 nm; (**c**) images of commercially printed BC/MXene films; (**d**) images of colored BC/MXene films; (**e**) measured weight (mg) of BC/MXene polymers (diameter d = 4 cm); (**f**) BC/MXene films thickness (μm). Reprinted with permission from [26] 2021 Elsevier.

BC/MXene films, as shown in Figure 7a, maintain good transparency despite the presence of dark MXene nanolayers, with transparency decreasing as filtration time increases (Figure 7b). This quality makes them promising materials for electronic shielding. Moreover, BC/MXene films offer the convenience of 3D printing and can exhibit intricate patterns (Figure 7c). They can also be painted in various colors, enhancing their versatility (Figure 7d). These films are notably thin (1 to 2 μm) and lightweight (3.0 to 3.6 mg) (Figure 7e,f), making them attractive for microelectronic devices compared to other materials like aerogels. The combination of transparency, flexibility, and light weight positions BC/MXene films as promising candidates for various wearable and electronic applications [26].

**Figure 8:**
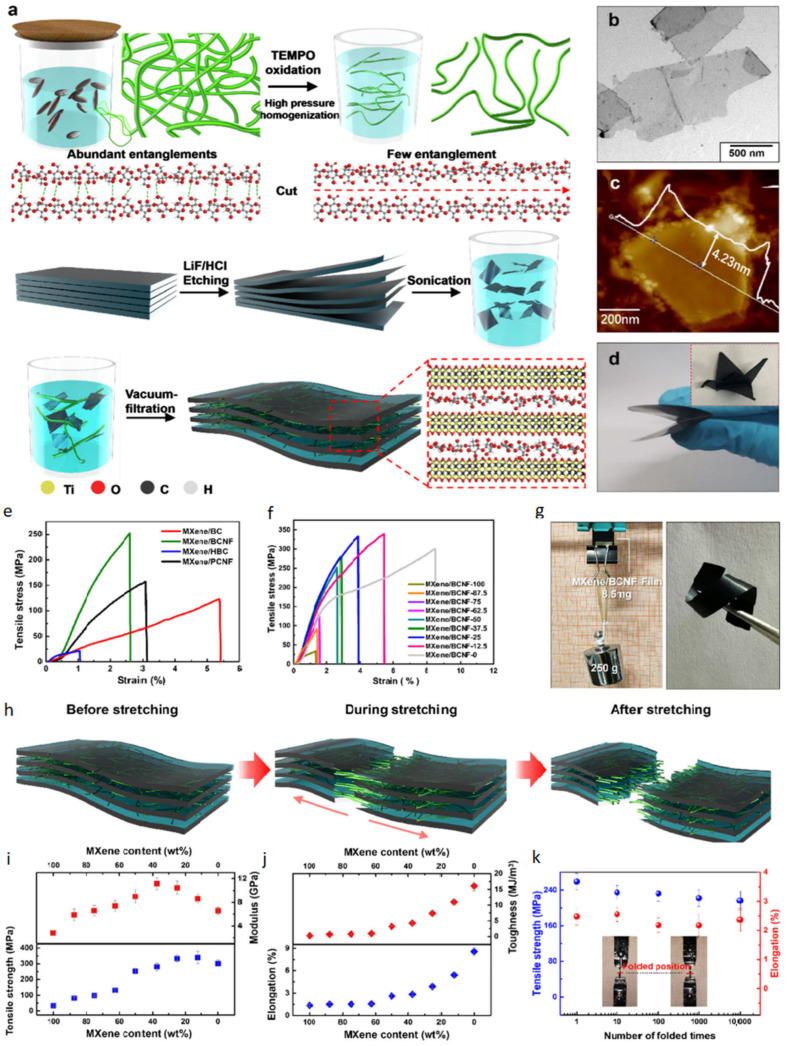
(**a**) illustration depicting the manufacturing process of the MXene/BC polymeric system; (**b**) TEM image of a pure MXene; (**c**) AFM image of a pure MXene; (**d**) image showcasing a folded MXene/BC composite film and a small paper crane created by folding the composite film; (**e**) strain-stress graphs of MXene/BC 50, MXene/B 50, MXene/HBC 50, and MXene/PCNF 50 films; (**f**) strain-stress graphs of the BC film, the MXene film, and MXene/BC 12.5–87.5 composite films; (**g**) images of the MXene/BC 50 film that exhibits ultra-flexibility and has the ability to withstand a weight of 250 g; (**h**) scheme of stretching process; (**i**) ultimate strength (red) and Young’s modulus (blue) of MXene/BC–0…100 composites; (**j**) elongation (blue) and impact strength (red) of MXene/BC–0…100 composite films; (**k**) tensile strength (blue) and relative elongation (red) of the folded MXene/BC 50. Adapted from [28] 2021 Springer.

Figure 8a illustrates the cultivation of BC using a dynamic fermentation process, resulting in the typical three-dimensional interconnected conformation of the BC structure [74,75]. Strong interactions between fibers can contribute to effective mechanical enhancement [76] but can also lead to aggregation during vacuum filtration. Aggregation can reduce contact resistance and lead to the formation of densely stacked MXene/BC layers. A gentle TEMPO oxidation treatment was conducted to achieve the desired outcome, followed by high-pressure homogenization. The AFM image confirms that the resulting BC maintains a well-designed structure with an extremely low (30–50 nm) thickness. This thinness is crucial in improving the mechanical properties and reducing the contact resistance between MXene layers. The synthesis of MXene involved the selective etching of Ti_3_AlC_2_ using a mixture of HCl and LiF. The resulting etched powder was manually stratified through shaking to create a stabilized dispersion. Morphological studies depicted in Figure 8b and c reveal the exfoliated MXene nanolayers with a significant size of 4.23 nm. The resulting MXene/BC composite film displayed remarkable flexibility and foldability, as shown in Figure 8d [28].

The MXene/BC composite film demonstrated high tensile strength (81.5 ± 9.2 MPa), elastic modulus (9.0 GPa), elongation (2.6%) and impact strength (3.2 MJ/m^3^) (Figure 8f). In addition, the data presented in Figure 8g shows that the composite film is capable of supporting approximately 30,000 times its own weight and can withstand multiple folds without tearing [28].

It should be noted that during the stretching process, the MXene plates are destroyed in a zigzag pattern, and the use of BC leads to an increase in elongation and an increase in the strength of the MXene/BC composite film [28].

**Figure 9:**
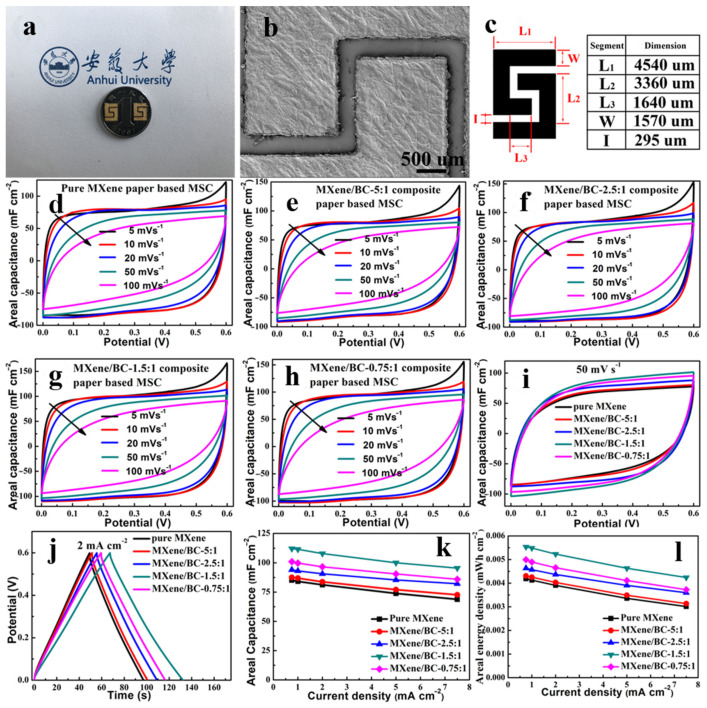
(**a**) picture showcasing standardized micro-supercapacitor (MSC) blocks made with MXene/BC composite; (**b**) the SEM image from equivalent MSC electrodes; (**c**) dimension sizes of electrodes of standard MSCs based on MXene/BC with mass ratio ranging from 5:1 to 0.75:1, and clean MXene paper (scan rates 5–100 mV∙s^−1^); (**d**) paper MXene-based MSC; (**e**) MXene/BC 5:1-based MSC; (**f**) MXene/BC 2.5:1-based MSC, (**g**) MXene/BC 1.5:1-based MSC; (**h**) MXene/BC 0.75:1-based MSC; (**i**) current–voltage characteristics measured at scan speed of 50 mV∙s^−1^; (**j**) galvanostatic charge–discharge (GCD) curves of the devices analyzed at the specified current density (2 mA·cm^−2^); (**k**) the evolution of flat capacity depending on the current density; (**l**) area energy density against the current density. Adapted from [77] 2019 John Wiley and Sons.

MXene/BC composite films have been incorporated into standard planar stacks of micro-supercapacitors using laser cutting technology. The mass fraction of BC fibers has been shown to influence the electrochemical performance of MXene/BC composite films. For comparison, neat MXene films and MXene/BC composite films with different mass fractions of BC fibers were analyzed (Figure 9).

When comparing the MSC blocks, it was observed that as the mass fraction of BC fibers increased, the peak current density of the corresponding blocks showed a noticeable increase at the same sweep speed [77].

The inclusion of BC fibers resulted in an augmentation of the devices’ flat capacitance and energy density. MSC block based on MXene/BC composite film with a weight ratio of 1.5:1 exhibited the largest capacitance (112.2 mF∙cm^−2^) and an energy density (0.00554 mWh∙cm^−2^) value [77].

**Figure 10:**
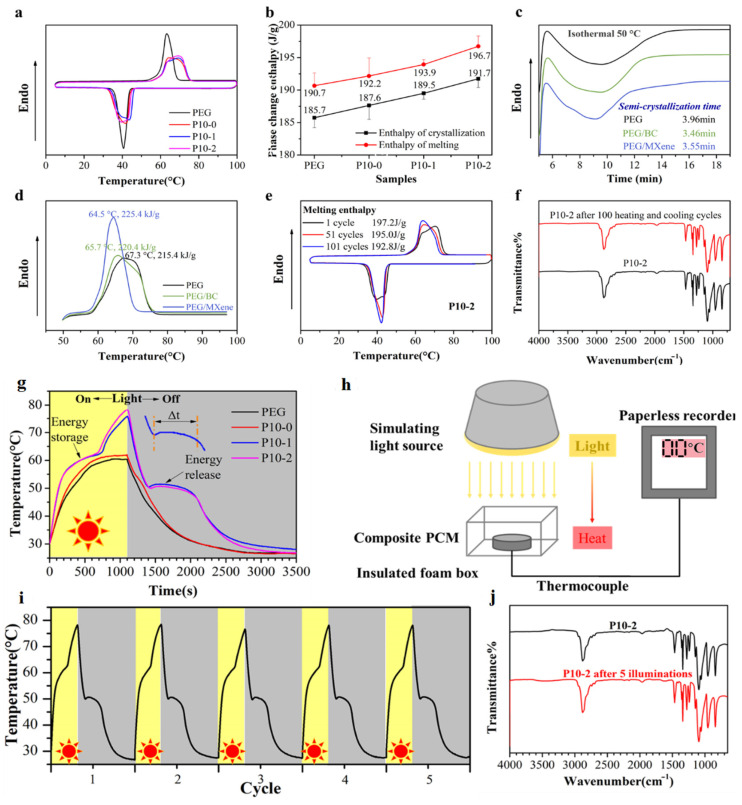
(**a**) Heating–cooling curves of clean PEG and composite PCMs P10-n measured using differential scanning calorimetry (DSC) method; (**b**) phase transition enthalpy of clean PEG and PCM P10-n; (**c**) time of semi-crystallization for PEG, PEG/BC, and PEG/MXene that was isothermally crystallized at temperature of 50 °C; (**d**) melting curve analysis and Δ*H_m_* values for PEG, PEG/BC, and PEG/MXene composite after crystallization; (**e**) DSC P10-2 heating–cooling curves after 100 cycles; (**f**) corresponding FTIR after 100 heating–cooling cycles; (**g**) temperature curves of pure PEG (630.2 mg) and PCMs P10-0 (628.7 mg), P10-1 (641.7 mg), P10-2 (611.3 mg) composites; (**h**) experimental configuration for converting light into heat; (**i**) thermal curves for P10-2 after 5 cycles; (**j**) corresponding FTIR spectra after 5 photothermal cycles. Reprinted with permission from [78] 2019 Elsevier.

The thermal energy storage capacity is an important characteristic of phase change materials (PCMs). However, the incorporation of non-phase transition functional materials in traditional composite PCMs results in a significant reduction in their phase transition enthalpies [78,79,80]. The content of each component in the samples was determined using thermogravimetric analysis (TGA), and the P10-2 sample exhibited a PEG loading of 97.9 wt.%, which maintained a significantly high energy storage density. The DSC curves of both pure PEG and P10-n composites are depicted in Figure 10a.

Table 2 [78] presents additional phase transition performance data for PEG and P10-n composite PCMs, such as melting/crystallization temperature (*T_M_/T_C_*) and melting/crystallization enthalpy (Δ*H_M_*/Δ*H_C_*).

Unexpectedly, the melting enthalpy of clean PEG, P10-0, P10-1, and P10-2 were 190.7 J/g (100%), 192.2 J/g (100.8%), 193.9 J/g (101.7%), and 196.7 J/g (103.1%), correspondingly; while Δ*H_C_* of them were 185.7 J/g (100%), 187.6 J/g (101.0%), 189.5 J/g (102.0%), and 191.7 J/g (103.2%), accordingly (Figure 10b). The phase change enthalpy was proven to increase in PCMs with the introduction of small amounts of BC aerogels and MXene photothermal conversion materials. BC and MXene promote the crystallization of PEG (Figure 10c,d). Figure 10e shows the possibilities of long-term use of PCM. The thermal stability and chemical resistance of the PCM were also proved (Figure 10f). PCMs containing MXene have demonstrated outstanding photothermal conversion capabilities (Figure 10g) and a high heating rate (Figure 10h) [78]. Thus, PCMs demonstrate high results, which makes them potentially suitable for storing solar energy.

The structural characteristics of composites based on bacterial cellulose and MXene are summarized in Table 3.

Due to flexibility, high sensitivity, electrical conductivity, ultrathinness, high energy storage capacity, and high energy storage capacity, these fundamentally new composites are beginning to be used in various fields of human activity, the main directions of which are outlined in the next section.

## 4. Application of BC/MXene-Based Composites

The unique properties of the composite material based on BC/MXene make it possible to produce high-strength, flexible, lightweight, mechanically stable structures.

**Figure 11:**
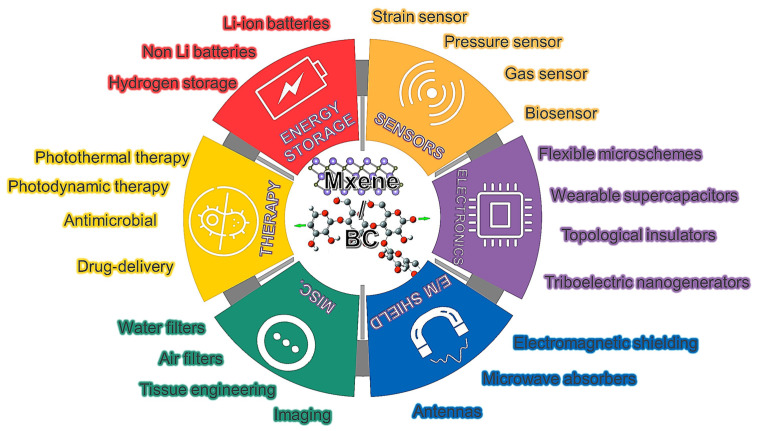
Schematic view of BC/MXene-based composite applications.

Owing to their excellent properties, these composite materials have promising applications in flexible electronics, smart electronic devices, energy storage, disease therapy, tissue engineering, electromagnetic interference shielding, etc. (Figure 11).

### 4.1. Sensor Electronics

Biocomposite-based sensors are widely used in many industries due to their excellent sensitivity, rapid frequency response, exceptional durability and reproducibility, and ease of fabrication [82]. Applied pressure causes a modification in the contact area of the sensitive material, resulting in an alteration to its electrical properties and the generation of a signal indicating changes in current resistance. In recent years, there have been advancements in the fabrication of different nanostructured or microstructured materials, aiming to achieve exceptional sensitivity characteristics [83,84,85]. Graphene nanosheets [86,87,88,89], metal nanowires [90], carbon nanotubes (CNTs) [91], polymers with high conductivity [92], and different new materials have been studied as promising materials to improve piezoresistive characteristics, for example, biomass carbon membranes [93] and conductive aerogels [88,94]. The authors designed a pressure sensor from graphene paper with a high sensitivity (17.2 kPa^−1^) and detection limit (10 Pa) [88]. A similar device was fabricated by Chen et al. from charred crepe paper with a detection limit of 0.9 Pa and sensitivity of 5.67 kPa^−1^ [95]. Despite significant advancements in the development of sensitive pressure sensors, the overall characteristics, such as low operating potential, high sensitivity, long-term stability, wide sensitivity range, and fast response time, still fall short of practical requirements [16]. The task of producing a flexible pressure sensor continues to be a challenge, primarily due to the need to design and fabricate sensor materials with intricate sensory capabilities. Furthermore, the internal atomic structure of conventional piezoresistive materials presents a hurdle in enhancing sensitivity, as the movement of atoms faces extreme resistance [5]. Taking this into account, studying new materials and structures with excellent sensory characteristics is at the forefront of creating high-performance wearable electronic pressure sensors [16].

MXenes are of great interest in electrical and chemical applications, namely batteries [12,13], supercapacitors [6,10,11], and catalysts [96] via their intrinsic conductivity and hydrophilicity [97,98]. MXene, which possesses distinctive properties such as adjustable conductivity resulting from variable interlayer spacing under specific pressure, shows promise as a potential candidate for manufacturing highly sensitive pressure sensors [5,99,100,101,102,103]. A pressure sensor was created using pure multilayer Ti_3_C_2_T_x_ based on MXene as a sensitive material, with a detection limit of 10.5 Pa and a measurement coefficient 180 [5]. In their research, Yu et al. introduced a flexible and degradable pressure sensor by affixing an MXene-impregnated wipe onto a biodegradable polylactic acid substrate. The sensor exhibited a sensitivity of 3.81 kPa^−1^, a detection limit of 10.2 Pa, exceptional reproducibility over 10,000 cycles, and a high capacity for decomposition [82]. Nonetheless, a few challenges must be addressed in MXene-based flexible pressure sensors. These include ultrahigh intrinsic conductivity and a restricted compression ratio of the interlayer space in MXene, resulting in a high initial current value. Consequently, this minor change in the conductive path at low-pressure loads restricts the sensitivity of piezoresistive sensors [82]. The detection limit and sensitivity play a crucial role in determining the potential applications of a pressure sensor, especially in measuring delicate human biological activities or utilizing it as electronic skin. Therefore, a rational approach to meet these requirements is to develop an MXene pressure sensor with an innovative nano-microstructure that combines a high level of compression in the interlayer space and sufficient mechanical strength.

BC, a commonly employed cellulose, is composed of interconnected ultrathin nanofibers with abundant oxygen-containing functional groups within its polymer chains. These characteristics make BC an exceptional pressure buffer, as it possesses high tensile strength and favorable hydrophilicity due to spontaneous interaction in hydrogen bonds [103,104,105]. In their study [57], Chen et al. created a carbon aerogel sensor by annealing a composite of BC and MXene. The sensor demonstrated exceptional detection capabilities, particularly in terms of small detection limits and response–recovery time. Such detection ability can be attributed to the minimal alteration in the interlayer space between the carbonized carbon derived from BC and MXene. It was reported by Yang et al. MXene/BC pressure sensor has a high sensitivity (95.2 kPa^−1^, 50 Pa) and a fast response rate (95 ms), along with excellent mechanical stability (25,000 cycles) [81]. The addition of BC offers a highly flexible buffer that enhances the separation of MXene layers. Through hydrogen bond interactions, it imparts excellent material integrity to the hybrid film, resulting in a high mechanical strength [97]. The sensor can be affixed to different areas of the human body to monitor various health conditions, such as pulse and heartbeat for slight pressure, acoustic vibrations, and large flexion movements like arm flexion and knee movement. Additionally, it can serve as an interactive spatial pressure distribution sensor, showcasing its promising potential in human–machine communication, electronic skin, and medical applications.

### 4.2. Tissue Engineering

The process of healing skin wounds involves a series of intricate steps, including blood clotting, inflammation, cell growth and tissue formation, and the formation of new skin cells [35]. Enhancing wound healing requires the development of a wound coating that serves as a protective barrier, while also promoting the regeneration of skin tissue for optimal healing [106]. According to Mao et al., the application of a hydrogel bandage was found to expedite the wound healing process by creating a moist environment and absorbing excessive exudate. This bandage is also easily removable without causing any harm, and it provides a cooling effect on the wound surface, thereby alleviating pain for the patient [107,108]. In many cases, bandages are primarily designed to improve tissue rehydration and prevent infections in the wounded area. However, they cannot often actively influence the behavior of endogenous cells for effective wound healing, resulting in more passive healing [109]. Disrupting multiple biological pathways and the inflammatory response can lead to wound healing failure due to uncontrolled modulation of the healing process [23]. Authors [106] created a new approach in therapy enhancing the regenerative activity of skin and tissue injuries. When the epithelium is damaged, a naturally occurring endogenous direct current electric field is generated due to the transepithelial potential (TP) differences. The electric field persists at the site of the wound until the healing process is fully completed [24,110]. The physiological electric field generated in this way can act as a significant signal that controls various cell behaviors crucial for injury healing and tissue repair. Cellular adhesion, cell division, differentiation, and migration are among the activities that play a major role in the regenerative activity [110,111,112]. Mao et al. [35] used electrical stimulation to heal skin faster. Electrical stimulation works like the natural healing process in the human body. Studies have demonstrated that ES is beneficial for wound healing and skin tissue regeneration. It has been shown to enhance angiogenesis, improve blood circulation, prevent oedema formation, reduce inflammation, and promote granulation tissue formation and collagen synthesis [106,113,114,115,116,117]. Moreover, electrical stimulation induces skin wound re-epithelialization due to the migration of fibroblasts, keratinocytes, and epithelium cells [118,119,120,121]. Depending on the place, the conductivity of skin varies from 2.6 to 1 × 10^−4^ ms∙cm^−1^, which is quite high [19,20]. Nevertheless, most bandages, including hydrogel wound bandages, lack electroactivity and cannot respond to physiological electrical signals or external ES during the healing process at wound sites. To tackle this issue, researchers [106] devised an innovative electroactive hydrogel bandage that can effectively respond to ES and enhance the wound healing process. Bacterial cellulose has unique properties, including high water absorption and mechanical strength, good permeability and biodegradability, and favorable biocompatibility [122,123]. The impressive characteristics of this material hold great promise for its use as a wound bandage or artificial skin substitute [124,125,126,127]. The main problem is that BC is not conductive, aggravating wound healing regulatory functions.

Functional nanoparticles are employed to enhance the electrical conductivity and mechanical properties of hydrogels [128]. Among most nanomaterials, MXene (Ti_3_C_2_T_x_ system) has attracted material scientists’ attention due to its high mechanical, biological and electrical properties [129]. The exceptional characteristics of BC and MXene combinations have led to the development of various biodegradable and electroactive regenerated BC/MXene hydrogels with different Ti_3_C_2_T_x_ compositions. According to Mao et al. [35], these biodegradable and electroactive BC/MXene hydrogels hold great promise as skin wound healing dressings. The electroactive properties of these materials can enhance proliferation by facilitating the transmission of electrical signals. NIH 3 T3 cells have been shown to accelerate the process of wound healing.

**Figure 12:**
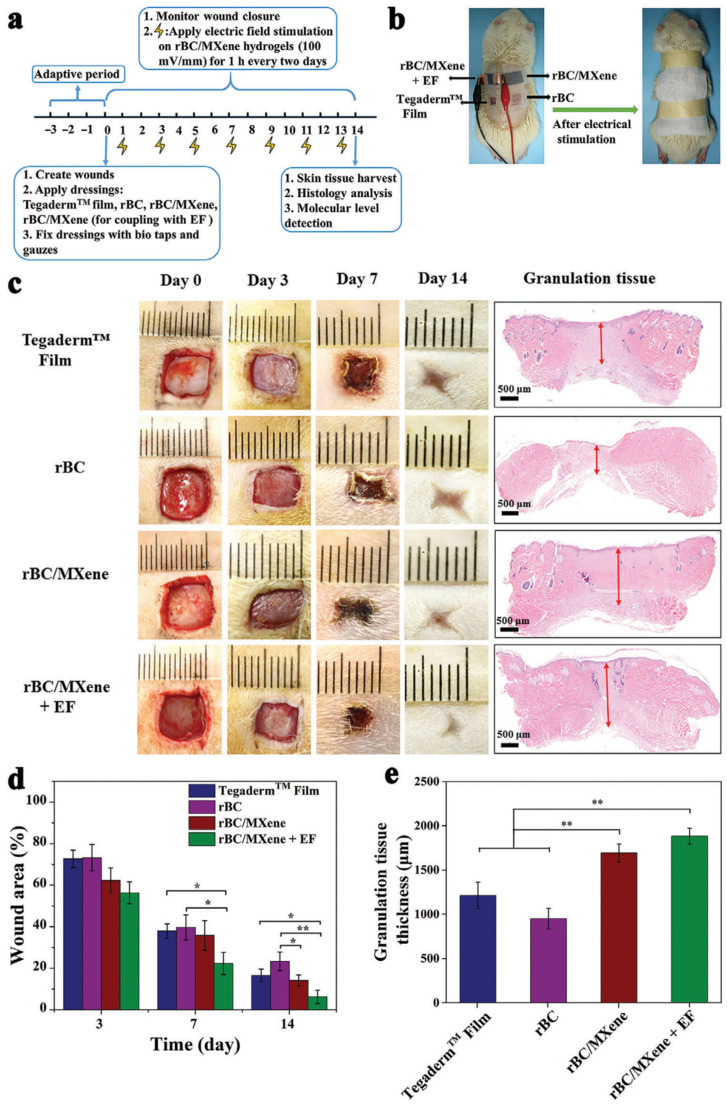
(**a**) Illustration of in vivo wound healing experiment; (**b**) an image of various wound healing bandages, namely Tegaderm commercial film bandage (control), BC/MXene hydrogel, and BC/MXene hydrogel with EF; (**c**) captured wounds after 0, 3, 7, and 14 days, as well as photographs highlighting granulation tissue at day 14 from various treated samples (granulation tissue marked by red arrow); (**d**) relationship between wound area and time for each sample; (**e**) thickness of granulation tissue at 14 day for each bandage. Data are depicted as mean ± SD (*n* = 4–6), and one-way ANOVA with Tukey’s multiple comparison test was applied for the analysis of significant difference. * *p* < 0.05, and ** *p* < 0.01, as compared among all the groups. Reprinted with permission from [35] 2020 John Wiley and Sons.

This research [106] confirmed the efficiency of BC/MXene 2% hydrogel for wound healing use. Furthermore, the effectiveness of wound healing was enhanced by applying electrical stimulation (ES). To examine the synergistic impact of BC/MXene and ES hydrogel bandages on wound healing, electroactive BC/MXene hydrogel bandages were connected to a DC source, allowing for the application of an external electric field (EF) (Figure 12a,b). Specific adverse effects were observed as a result of prolonged exposure to electric fields (EF) [90,117]. Hence, in order to achieve a favorable balance between wound healing rate and the strength of the electric field, a range of electric fields from 0 to 400 mV∙mm^−1^ were applied between copper electrodes on hydrogel rBC/MXene bandages. The findings indicated that as the force of the electric field (EF) increased to 300 mV∙mm^−1^, wound healing accelerated compared to the control group. When wounds were treated with BC/MXene hydrogel bandages along with an EF of 100 mV∙mm^−1^, the wound area after 12 days of treatment was considerably smaller than in any other group. On the contrary, when the EF reached 400 mV∙mm^−1^, wound healing was notably slower, and the wound area was the biggest. After applying EF of 100 mV∙mm^−1^, the macroscopic appearance of the wounds under various treatments on days 0, 3, 7, and 14, respectively, was quantitively measured, as depicted in Figure 12c. The wound area was reduced consistently across all groups over time, following the same pattern at various time intervals: rBC/MXene + EF group < rBC/MXene hydrogel group < Tegaderm film group < rBC hydrogel group. When electrical stimulation (ES) was applied, wounds treated with rBC/MXene hydrogel bandages (rBC/MXene + EF group) exhibited the smallest wound area compared to all other groups at any given time (93.8% ± 3.2% closure). According to the results presented in Figure 12d, the wound treated with rBC/MXene + EF exhibited a significantly smaller size compared to the group treated with Tegaderm commercial film and the group treated with erythrocyte hydrogel at days 7 and 14, respectively. Furthermore, a notable decrease in wound size was observed in the rBC/MXene group without electrical stimulation compared to the rBC hydrogel group on day 14. These findings suggest that the electroactive rBC/MXene hydrogel has the potential to enhance the healing of skin wounds, primarily due to the influence of MXene. Additionally, the application of concurrent electrical stimulation (ES) can further enhance the acceleration of the healing. Multiple published papers support this hypothesis. Integrating electroactive materials into the bandage membrane or hydrogel and applying electrical stimulation greatly expedited wound healing [118,119].

### 4.3. Supercapacitors

Currently, numerous deformable energy storage systems are in the development stage. Extensible supercapacitors (SCs) have demonstrated significant promise as suitable deformable sources. This path of application is available primarily due to their impressive attributes such as long lifespan, safety, high density and power, quick recharge time, etc. [77,130,131,132]. Traditional extensible supercapacitors utilize multilayer electrodes to achieve their extensibility. It could be accomplished by applying prestress to the elastic substrate before assembling the components, as well as the extension ability of the wavy electrodes [132]. Although wave-shaped multilayer electrodes offer appealing advantages such as high surface capacitance and a straightforward manufacturing process, integrating them into planar tensile electronic circuits is often challenging. Furthermore, these wave-shaped components tend to detach from each other during repeated tensile deformation, resulting in a decline in electrochemical performance. On the other hand, the emerging 2D planar expandable arrays of MSCs are more desirable to integrate into expandable electronics. They have segmented structural design that demonstrates a higher level of rationality [133,134,135]. As MSC islands with planar electrode configuration function solely as active regions for energy storage and are not subject to tensile strain, they enable interdigital electrodes to maintain a consistent distance. The proper distance prevents them from breaking under strain, ensuring the electrochemical stability of the device. The arrangement of MSC islands with a 2D planar interdigital transducer (IDT) can minimize the distance for ion diffusion, leading to an enhanced device power density. At the same time, the device gains remarkable stretchability due to an elastic and conductive framework connecting these islands. Of greater significance, these MSC islands have the flexibility to be grouped and interconnected as desired using an elastic frame. This enables precise control over the output voltage and current density to meet specific needs [136,137]. Hence, there is a significant demand for the development of a simple and effective approach to fabricate deformable MSCs that possess high surface capacitance, exceptional stretchability, excellent integration capability, and electrochemical stability. However, achieving this goal is a challenging task.

Typically, the surface capacitance of a supercapacitor device is proportional to the specific capacitance of the electrode materials employed [138,139,140]. The most direct and effective approach to enhance their surface capacitance, which are typically based on conventional carbon materials, is to utilize a new electrode material with a high specific capacitance for the design and fabrication of MSC islands. Because of the unique two-dimensional layered structure and metallic conductivity of MXene [12,141,142,143,144,145,146,147,148], as well as the high specific volume capacity exhibited by these materials, they could potentially enhance the performance of planar MSCs [149,150,151,152,153]. There is a limited number of studies focused on stretchable MXene-based micro-supercapacitors (MMSCs) that possess both decent elongation and high areal performance. Furthermore, the repackaging of MXene nanosheets during the fabrication of electrode films poses a challenge in achieving complete penetration. To overcome this obstacle, interlaminar nanolayers consisting of 1D carbon nanofibers, like CNTs, are commonly utilized to facilitate film repackaging [154,155,156,157,158].

On the other hand, the future large-scale commercial utilization of these carbonaceous materials is greatly restricted due to their relatively high cost, low production rate, and the requirement for stringent control of their synthetic environment. In contrast, cellulosic fibers offer the advantage of being an environmentally friendly, renewable, and sustainable natural supply [159]. BC nanofibers can be synthesized completely pure natural substances without the need for special synthetic media or high-cost equipment. In [160], an MXene composite was fabricated from delaminated multilayers of Ti_3_C_2_T_x_ and BC nanofibers. The 2D MXene flakes were aligned in the planar direction to enhance the overlap area and minimize contact resistance. Additionally, the presence of 1D BC fibers can be observed to serve as a stabilizing agent, effectively binding the 2D MXene flakes in the composite film. This leads to a substantial improvement in the mechanical strength of the hybrid film. An essential aspect is that bacterial cellulose characterized by its nanofiber structure can function as an interlayer to prevent excessive stacking of fully delaminated MXene nanosheets. Additionally, it aids in regulating the interlayer spacing during the process of film formation. This regulation facilitates improved ion transport into the interlayer space and results in an increased affected area [160]. The flexible, tensile, and twistable solid-phase MMSCs were prepared using laser cutting due to outstanding mechanical stability and electrochemical performance of composites. They were characterized by a high specific capacitance of 111.5 mF/cm^2^ [160]. Tang et al. created hybrid aerogels by incorporating BC and MXene into a PEG matrix [78]. The composite material that is formed exhibits exceptional energy storage capacity, remarkable shape stability (maintained even at temperatures as high as 120 °C), and photothermal conversion capability.

### 4.4. Electromagnetic Interference Shielding

In the past few decades, the prevalence of wireless communication and electronic devices has increased significantly as a result of economic progress. However, this rapid technological advancement in telecommunications and the widespread use of electronic devices has brought about a significant issue: electromagnetic radiation. This electromagnetic interference (EMI) poses a threat to electronic equipment, negatively impacts human health, and can lead to severe diseases [27,161,162]. Conventional EMI shielding materials have traditionally consisted of metals and metal composites, primarily chosen for their excellent electrical conductivity. However, their drawbacks, including high density, limited corrosion resistance, and elevated costs, have hindered their extensive utilization in modern mobile electronics [27,161,162,163]. Over the past few years, there has been a surge in the development of carbon materials, such as CNTs or graphene, as conductive nanofillers for polymer composites. These composites are specifically designed to serve as materials for shielding against electromagnetic interference [2,164,165,166,167,168]. Carbon polymer composites possess numerous noteworthy advantages, demonstrating their superiority. These advantages include their environmentally friendly nature, low density, exceptional flexibility, and remarkable chemical stability [27,161,162]. In many applications, the thickness requirement of carbon–polymer composites, typically in the millimeter range, is often impractical for achieving satisfactory interference shielding performance. This is precisely why the emergence of 2D inorganic conductive materials, such as MXene [62,64], holds great significance these days. MXene could possibly become an alternative to widespread materials based on carbon and metals [63,169,170]. A groundbreaking study by Shahzad et al. highlighted the exceptional EMI shielding efficiency of MXene films, achieving an impressive 92 dB with a mere 45 µm thickness. Since then, a multitude of MXene-based materials have been documented for their effectiveness in EMI shielding applications [63,152,171,172,173]. Compared to pure MXene and MXene/inorganic composites, MXene/polymer composites display many advantages in terms of EMI protection. These advantages include low weight, excellent corrosion resistance, flexibility, and good processability [53,65,67,174]. There is limited research on polymers that can serve as matrices for MXene-based composite materials. For instance, authors [175] synthesized a 26 µm thick MXene/calcium alginate-based aerogel that showed *SSE/t* = 17,586 dB∙cm^2^∙g^−1^. Jin et al. made a MXene/PVA polymer composite with a maximum *SSE/t* = 9343 dB∙cm^2^∙g^−1^ [176]. Research on thin MXene/polymer composite films has demonstrated satisfactory electromagnetic interference (EMI) shielding characteristics, making them a promising candidate for ultrathin MXene/polymer composite films in EMI shielding applications. However, the mechanical properties of these composites have not been thoroughly investigated, and it is suggested that further reduction in thickness is possible, particularly for aerospace industry applications. Therefore, developing an effective preparation strategy is essential to produce thinner and stronger EMI shielding materials. BC, an organic material with excellent stability, controlled pore structure, and film-forming properties, is considered a highly viable matrix material for this purpose [6,26]. The paper [26] stated that the combination of BC and MXene would result in EMI shielding materials with exceptional efficiency and strength. They prepared a 3D porous MXene/BC composite by blending MXene and BC suspension to test this. The composite exhibited outstanding capacitive performance when used as an electrode for capacitors. In a recent study, the group led by Ma et al. successfully developed a vacuum-filtered Janus BC/MXene film that showed high EMI shielding efficiency [78]. However, using a fragmented BC destroyed the structure of a three-dimensional network, which reduced its mechanical properties. In our review article, we did not describe the use of nanoparticles of metals and their oxides [177] combined with BC/MXene. Still, it is worth pointing out that their inclusion in the NBC/MXene composite during the manufacturing of medical dressings and bandages increases the effectiveness of treating superficial wounds by significantly reducing the activity of bacteria. Such metals could be Au, Ag, Zn, Cu, ZnO, and Fe_2_O_3_ [178,179,180,181,182,183,184]. The research on these composite materials will be actively conducted soon.

It is also worth noting that BC and MXene can be used separately from each other to solve many applied problems in biology, medicine, energy, and sensors, for example, to increase the capacity of batteries, improve the absorption of electromagnetic signals, enhance the sensitivity and response time of sensors, etc.

### 4.5. Honorable Mention

This review article briefly described the preparation, properties, and applications of BC/MXene systems. Despite the relatively large amount of analyzed literature sources (186 articles), we cannot describe all possible applications of this promising composite material in detail. Firstly, we are strictly limited by the scope and size of the review article, and secondly, some applications of the considered nanocomposite material were not mentioned in the text of our article due to the relatively small number of publications devoted to them. That is why we decided to mention these possible applications in these conclusions.

For example, in work [185], very tiny signals of the movements of bending fingers, wrists, and heartbeat electric signals provided electronic transfer in MXene during the sensing process. That is why the authors of this paper propose a universal platform for the use of MXene in the manufacture of three-dimensional composite aerogels for high-performance flexible e-skin sensor systems. On the other hand, the three-dimensional interwoven Ti_3_C_2_/MXene networks fabricated using BC can be used as cathodes for rechargeable magnesium ion batteries with high capacity and cycling performance (capacity retention up to 88% after 100 full charge–discharge cycles) [186]. Large distances between layers, optimized diffusion paths, a high diffusion coefficient, and a large amount of magnesium contribute to such good electrochemical characteristics.

In work [177], the authors tried manufacturing composites with a nacreous lamellar structure. They created a film consisting of MXene modified by polydopamine and nanofibers from bacterial cellulose. These nanocomposites demonstrated improved properties as an activator of controlled devices. The activator has excellent strength, tensile strength and impact toughness (15.3 MJ/m^3^), and conductivity (up to 2848 Ohm/cm). In addition, this nanocomposite is very sensitive to moisture and has a fast response time (1.6 s) and a large initial actuation force. Additionally, it has been shown that this material can work as an electrical switcher, a robotic lever, or a stepper motor. The publication’s authors believe that their work overcomes the shortcomings of existing MXene-based actuators and lays the groundwork for their further use as moisture-controlled devices.

The problem of powering implanted medical devices (IMD) deserves special attention since a living organism requires biocompatible, stable, and miniature sources of electricity. Engagement in the salinity gradient presents an appealing and effective method of generating electrical current. At the same time, a negatively charged bacterial ion channel mimetic is usually used. The nano biocompatible NBC/MXene membrane is used as an osmotic energy generator. Using NBC fibers in MXene nanolayers adds space charge and significantly increases the flow of ions. From the point of view of in vivo use, the salt gelatin hydrogels are being utilized as electrolytes in an unprecedented manner. The effect of the combination of 1D nanorods and 2D MXene nanolayers allows for obtaining an energy concentration of up to 2.58 W/m, which significantly exceeds the concentration when using traditional solid electrolytes. In vivo and in vitro studies demonstrate good biocompatibility of NBC/MXene hybrid membranes. The potential of NBC/MXene-based membranes for power sources in medical devices is attributed to their highly efficient osmotic energy conversion and excellent biocompatibility.

## 5. Conclusions

Hydrogels that integrate the unique properties of MXene and the inherent polymer characteristics of bacterial cellulose exhibit substantial promise in various applications, including sensor electronics, electromagnetic shielding, solar energy storage in supercapacitors, tissue engineering, wound healing, and related fields. With its exceptional mechanical properties, electrical conductivity, and biodegradability, it is highly sought after for biomedical purposes. Additionally, MXene offers significant potential for surface modification and functionalization thanks to its abundant surface-encapsulating functional groups such as hydroxyl, fluorine, and oxygen. By varying the composition of MXene and polymer, tuning mechanical flexibility and electrical conductivity for each practical application is possible. The impact of MXene concentration is still in the initial stages of investigation. However, recent findings indicate that enhancing the composite’s properties can be accomplished by optimizing its content. In this review, fabrication, characteristic features and future perspectives were discussed.

BC/MXene composites are improving at treating and diagnosing different diseases, which is very encouraging. Nevertheless, in order to grant access to their clinical application, there are nonetheless numerous obstacles that require addressing. One of the main challenges in using BC/MXene for tissue engineering is the lack of standard protocols that all scientists for safety testing must follow. We currently see different studies using different cell cultures, doses, and/or animal models. In addition, toxicology must be performed for healthy and diseased models. More efforts should be made to increase the low yield of MXene in scaleup production to help MXene become more clinically useful in the biomedical field. Moreover, as previously mentioned, different bacterial cellulose and MXene composition types should be tested. Therefore, systematic studies are needed to determine which type of cellulose should be combined with MXene to understand hydrogel functional properties completely.

## Figures and Tables

**Table 1:** Mechanical properties of BC/MXene-based composites [26].

Sample	Tensile Strength (MPa)	Deformation at Break (%)	Viscosity (MJ/m^3^)	Young’s Modulus (GPa)	Number of Folding (Time)
BC	389.7 ± 5.3	10.1 ± 0.3	23.6 ± 1.4	6.6 ± 0.9	5055 ± 45
BX-1	407.3 ± 11.8	9.6 ± 0.2	24.3 ± 0.6	7.3 ± 0.3	5265 ± 87
BX-2	451.0 ± 19.2	9.6 ± 0.4	25.9 ± 4.4	7.6 ± 0.1	5560 ± 44
BX-3	469.4 ± 11.9	10.3 ± 0.4	28.3 ± 2.5	7.8 ± 0.7	5828 ± 65
BX-4	481.5 ± 7.3	10.8 ± 0.3	29.4 ± 0.2	8.0 ± 0.6	5944 ± 50
BX-5	532.9 ± 22.4	11.0 ± 0.9	31.1 ± 2.7	8.3 ± 1.3	6152 ± 51

**Table 2:** Differential scanning calorimetry data for pure PEG and PCM P10-n composite [78].

Sample	*T_M_* (°C)	Δ*H_M_* (J/g)	*T_C_* (°C)	Δ*H_C_* (J/g)
PEG	63.5 ± 0.3	190.7 ± 2.0	41.1 ± 0.4	185.7 ± 1.5
P10-0	67.5 ± 2.5	192.2 ± 2.8	39.7 ± 1.7	187.6 ± 2.1
P10-1	67.9 ± 2.7	193.9 ± 0.8	42.7 ± 0.8	189.5 ± 0.9
P10-2	67.0 ± 2.1	196.7 ± 1.6	41.5 ± 1.7	191.7 ± 1.3

**Table 3:** Structural characteristics of composites based on bacterial cellulose and MXene.

Type of Composite	The Composite Form	Synthesis Method	Specific Properties	Application	References
Ti_3_C_2_T_x_/BC	Film	Vacuum filtration	High mechanical strength (225 MPa), low detective limit (0.4 Pa), outperformed repeatability (25,000 cycles)	Pressure sensor	[16]
BCs/MXene film	Film	Vacuum filtration	Flexibility, transparency, conductivity	Wearable electronics, electromagnetic interference (EMI) shielding	[26]
rBC/MXene Hydrogel	Hydrogels	Chemical precipitation	Flexibility, electrical conductivity	Wound dressing	[35]
C-MX/BC-x carbon aerogel	Carbon aerogel	Directional freezing, freeze-drying, carbonization	Super compressibility, elasticity, high sensitivity (0–10 kPa)	Electronics and electronic skins	[57]
Ti_3_C_2_T_x_/BC	Film	In situ biosynthesis	Ultrathin films, electrically conductive, mechanically strong	Electromagnetic interference (EMI) shielding	[27]
BC/MXene hybrid aerogels	Hybrid aerogels	Freeze-drying	Ultralight, excellent shape stability, high energy storage capacity	Solar thermal energy storage	[78]
MXene/BCNF	Film	Vacuum filtration	High tensile strength (252.2 MPa), excellent folding endurance (10,000 times), high electrical conductivity (443.5 S cm^−1^).	Electromagnetic interference (EMI) shielding	[28]
MXene/BC	Paper	Vacuum filtration	Flexibility, mechanical strength	Micro-supercapacitor	[77]
MXene/BC derived	Carbon aerogel	Thermal annealing/carbonization	High sensitivity (95.2 kPa^−1^, 50 Pa)	Pressure sensor	[81]

## Data Availability

Not applicable.

## Supplementary Materials

The following supporting information can be downloaded at: https://www.mdpi.com/article/10.3390/polym15204067/s1, Figure S1: XPS survey spectrum of the pure d-Ti3C2Tx paper and d-Ti3C2Tx/CNF (50 wt %) composite paper.
