# Supplementary material for: Synthesis, Properties, and Applications of Nanocomposite Materials Based on Bacterial Cellulose and MXene
Source: Polymers (Basel). 2023 Oct 12;15(20):4067. doi: 10.3390/polym15204067 (PMC10610809; doi:10.3390/polym15204067)
Supplement: Supplementary file 1 [file polymers-15-04067-s001.zip › polymers-2528513-supplementary.pdf]

Supporting Information

Synthesis, Properties, and Applications of Nanocomposite Materials Based on Bacterial Cellulose and MXene

Aizhan B. Talipova [1], Volodymyr V. Buranych [2,3,*], Irina S. Savitskaya [1], Oleksandr V. Bondar [2], Amanzhol Turlybekuly [4,5] and Alexander D. Pogrebnjak [2,3,6,*]

[1] Department of Biotechnology, Al-Farabi Kazakh National University, Almaty 050040, Kazakhstan; talipova.aizhan@gmail.com (A.B.T.); irina.savickaya@kaznu.edu.kz (I.S.S.)
[2] Department of Nanoelectronics and Surface Modification, Sumy State University, 40000 Sumy, Ukraine; oleksandr.v.bondar@gmail.com
[3] Faculty of Materials Science and Technology in Trnava, Slovak University of Technology in Bratislava, 917 24 Trnava, Slovakia
[4] National Laboratory Astana, Nazarbayev University, Astana 010000, Kazakhstan; aturlybekuly@gmail.com
[5] Aman Technologies, LLP, Astana 010000, Kazakhstan
[6] Faculty of Mechanical Engineering, Lublin University of Technology, 20-618 Lublin, Poland
* Correspondence: v.buranich@phe.sumdu.edu.ua (V.V.B.); a.d.pogrebnjak@gmail.com (A.D.P.); Tel.: +421-918305994 (V.V.B.); +380-662-376-564 (A.D.P.)

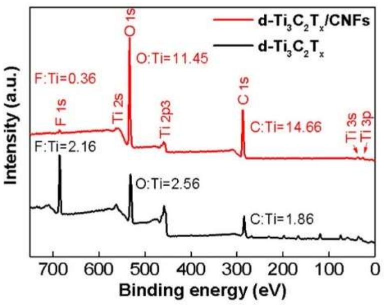

Figure S1. XPS survey spectrum of the pure d-$Ti_3C_2T_x$ paper and d-$Ti_3C_2T_x$/CNF (50 wt %) composite paper [53].

53. Cao, W.-T.; Chen, F.-F.; Zhu, Y.-J.; Zhang, Y.-G.; Jiang, Y.-Y.; Ma, M.-G.; Chen, F. Binary Strengthening and Toughening of MXene/Cellulose Nanofiber Composite Paper with Nacre-Inspired Structure and Superior Electromagnetic Interference Shielding Properties. ACS Nano 2018, 12, 4583–4593, doi:10.1021/acsnano.8b00997.
